# Influence of Supervisors’ Fairness on Work Climate, Job Satisfaction, Task Performance, and Helping Behavior of Health Workers During COVID-19 Outbreak

**DOI:** 10.3389/fpsyg.2022.822265

**Published:** 2022-04-29

**Authors:** WenXin Wang, Ahotovi T. Ahoto

**Affiliations:** ^1^Department of Public Administration, Law School, Shantou University/Institute of Local Government Development, Shantou University, Shantou, China; ^2^School of Management, Jiangsu University, Zhenjiang, China

**Keywords:** supervisors fairness, work climate, job satisfaction, task performance, helping behavior, COVID-19

## Abstract

The need for supervisors to exhibit fairness was a key motivating tool for effective health service delivery during the initial stages of the COVID-19 outbreak. Nonetheless, the number of deaths and hospitalization was alarming health workers were actively working throughout the time. This study explores the role of supervisors’ fairness in creating a work climate and job satisfaction that promote workers’ task performance and helping behaviors. The researchers adopted a quantitative method with a questionnaire used for data collection. SPSS and AMOS were used for data analysis, and statistical models of correlation and hierarchical regression were used to examine relationships among the variables. The study established that supervisors’ fairness has a positive effect on work climate, job satisfaction, task performance, and helping behavior of health workers. Work climate has positive effects of task performance and helping behaviors, whereas job satisfaction also has positive effects on employees’ task performance and helping behavior. The researchers recommended the need for supervisors to exhibit fairness to workers at all times and create room for the workers to appeal their decision to avoid the feeling of supervisors’ biasness.

## Introduction

The outbreak of COVID-19 in the later part of 2019 distorted many activities globally (Ros et al., 2021); nonetheless, all organizations were affected; the health sector carried the highest task of treating sick people no matter the level of threats (Stosic et al., 2021). Effective supervisory is vital to run an effective health system from the onset of the outbreak; supervisory creates the needed work climate and job satisfaction for employees to deliver their duties effectively (Alava and Guevara, 2021). Supervisor fairness have been established as a tool in reducing health workers’ turnover, hence influencing quality health delivery (Sakapas et al., 2019).

A recent research shows that coordination between supervisors and their subordinates is a significant determinant of the quality of service delivery in the healthcare sector (Mekhum and Jermsittiparsert, 2019). Many employees derive their motivations from their supervisors, which makes the actions and inactions of the supervisors pivotal to how employees settle in their work climate and the level of satisfaction they derive from their work (Back et al., 2020). A key quality expected from a supervisor is their ability to exhibit fairness. Supervisor fairness describes the ability of supervisors to apply the equal principle to workers in ways that show justice and equity by matching employees’ effort and commitment with their gains. Notable studies have drawn links between supervisors’ fairness, employees’ task performance and helping behavior. Nonetheless, these two tasks, performance and helping behavior, are important tools for organizational performance; the fairness of supervisors is pivotal in how they play out. Task performances measure the ability of employees to fulfill their daily tasks within the stipulated time and satisfactorily, whereas helping behavior examines the willingness of employees to accept extra duties when needed (Frye et al., 2020).

The principle of higher risked jobs matching higher gain is a fundamental incentive for people working in the risky environment (Karanikola et al., 2020); hence, with the COVID-19 outbreak increasing the risk faced by health workers, it was equally expected that the satisfaction they drive from the jobs opts to be higher than before (Xu et al., 2020). Creating a favorable work climate and job satisfaction at critical times remains the responsibility of supervisors since they are the leaders that workers look up to Aloisio et al. (2021). With the COVID-19 outbreak increasing burdens on health facilities and health workers due to increasing out-patients’ and in-patients’ attendance, health workers were going beyond their everyday tasks by offering helps to cope with the situation of the moment (Willemse et al., 2020). Another research determined that work climate and job satisfaction drive task performance and helping behavior. Workers’ perception about supervisors’ fairness influences the activities they engage in Lin et al. (2016).

Hence this study focused on the impacts of supervisor fairness in driving a good work climate and employees’ job satisfaction, helping behavior, and task performance within the healthcare institutions in Ghana.

## Literature Review

Impartiality in dealing with employees creates a sense of confidence in employees, knowing that hard work is appreciated with matching gains (Negarandeh et al., 2014). Every employee deemed it right to have access to a leadership that creates the feeling of having leadership support in every action they embarked on organizational interest (De Simone et al., 2018). At the same time, physical remunerations are very important to the workers; the psychological gain of getting respect and reward from leadership helps in building organizational citizenship (Kenny et al., 2016). Supervisors’ fairness in responding to employees’ lower friction between workers leads them to compete for appreciations based on the outputs rather than playing tricks to attract leadership attention (Kim et al., 2017). Jermsittiparsert and Burairak (2019) documented several studies that further established the point that the relationship between an employee and leadership plays a vital role in achieving quality health delivery.

The vital role of the supervisor–employees relationship remains topical in many social science pieces of research since work climate is built around this relationship (Fogarty et al., 2017). Numerous studies are finding ways by which this relationship can grow to drive the outputs of organizations (Nasir and Masek, 2015). Leader–Member Exchange Theory explicitly appreciates the coordination efforts of leaders in organizations to drive the visions by harmonizing employees’ efforts (Yang et al., 2009). Supervisors’ ability to apply the equal principle to employees without being subjective in dealing with employees must be judged from how employees perceived the supervisor, not through supervisors’ appreciation of their actions (Orgambídez-Ramos and Almeida, 2017). Supervisors in organizations possess authority, and they act between top-level leadership and employees; hence, their influence on employees is also driven by the perception of employees’ vulnerability to them (Chow et al., 2015). Supervisors’ fairness is a key to employees’ job satisfaction and work climate because promotion, increase in salary, job security, and others trickled down to the employees based on supervisors’ recommendations (Chow et al., 2015).

Supervisors’ fairness is driven from trustworthiness of the supervisors’ decisions (Nasir and Masek, 2015); being fair does not imply that supervisors’ decision should not have negative impact on employees (Kim et al., 2017). It is expected that there should be objective reasons behind supervisors’ decisions or actions explaining why a particular outcome is not favorable to sections of employees; this prevents the expression of the bad inner feeling that affects employees and makes them to question why (Aloisio et al., 2021).

The COVID-19 pandemic puts more responsibility for fairness in handling employees in matters of formation of teams, positioning workers at the front line, and handling other critical aspects of health delivery (Aloisio et al., 2021). While the fairness of supervisors drives home satisfaction among employees in critical times of outbreaks, unfairness increases employees’ anxiety (Jones et al., 2021). A previous study has shown that supervisors’ fairness builds organizational citizen, promoting task performance and helping behaviors of workers by building a sense of belongingness in employees (Zhang T. et al., 2021). However, impartiality can equally drive some employees to deliver their tasks, but employees who felt unevenly treated may not show passion for working (Fogarty et al., 2017).

### Work Climate, Enabling Factor in Task Performance, and Helping Behaviors of Workers

The work climate, work environment, job perception, and organizational climate are mostly substituted for one another (Orgambídez-Ramos and Almeida, 2017). Work climate defines the conditions within the working space that inspire workers’ behavior, perception, and feelings of being part of the organization (Bienengräber et al., 2021). Work climate creates the interplay of various levels of hierarchies of authority and how workers engage themselves (Baltes et al., 2009). Critically, work climate is not a physical, but a psychological, image that an employee creates in their mind about how trustworthy their engagements are (Bienengräber et al., 2021). Work climate helps employees to improve the social contract they build with the organization and the length at which they can stand for the organization even beyond their routine tasks (Teo et al., 2019).

Work climate was topical around the year 1974 as amplified by James and Jones research, which measured job characteristics, role characteristics, leadership characteristics, social characteristics, and organizational attributes. Parker, in 2013, further continued the discussion on work climate using role, job, supervisor, and organization attributes as the measuring tools (Chaves and Santos, 2016). Many other studies have explored that work climate that focuses on supervisors is a part of the creation or influencers of climate that exists at workplaces (Bienengräber et al., 2021).

Empirically, health leadership was called to action during the COVID-19 outbreak; the role of supervisors in creating the needed climate that can influence task performance and helping workers’ behaviors was paramount. Nonetheless, other actions contribute to the design of work climate; the role of the leader, in this case, the supervisor, is the driver of the actions of the subordinates by creating the perception of standing for them anytime they call and giving equal opportunities to all of them (Xu et al., 2020).

### Job Satisfaction, a Driver of Task Performance, and Helping Behavior of Workers

Job satisfaction is the feeling of deriving gratification from employees from their jobs. Job satisfaction may be summed up as the primary motivation behind one’s desire to work (Kenny et al., 2016). Though job satisfaction is universally determined by skills, job, longevity, and others, the studies show that absolute satisfaction is an individual affair (Khomami, 2018). The individual perception about satisfaction is why persons receiving lesser material and monitory value for their effort feel gratified than a person taking higher physical remunerations. However, in quantitative terms, job satisfaction is measured chiefly with physical attributes (Pung et al., 2017).

Job satisfaction became a crucial issue in organizational performance, prompting many scholars to come out with ways to measure and enhance employees’ satisfaction. Notable works ranged from Spector in 1985, Singh and others in 1996, to Bolton in 1997. These three studies agreed that supervisors play a crucial role in influencing job satisfaction; hence, supervisory roles run through all the indicators they designed for measuring the subject (Orgambídez-Ramos and Almeida, 2017). Satisfied employees deliver quality services as they sometimes go beyond their mandatory task performance, and helping behavior (Dirlam and Zheng, 2017).

At critical times of the COVID-19 outbreak, frontline health workers desired further motivations to attain satisfaction as their work was beyond normal health delivery activities (Xu et al., 2020). Knowing the risks associated with the job of healthcare delivery during the outbreak, supervisors and other healthcare authorities around the globe increased benefit packages to influence task performance and help behaviors among health workers (Liu and Lin, 2021).

### Hypotheses Development

Recent literature has established the relationship between supervisor fairness, work climate, and job satisfaction. Supervisors’ fairness, which in some cases is referred to as supervisors’ justice, positively influences work climate and job satisfaction (Orgambídez-Ramos and Almeida, 2017). Job satisfaction and work climate have been shown to influence employees’ task performance and helping behavior (Baltes et al., 2009). Studies that focused on organizational performance put a lot of responsibilities on supervisors by seeing them as the closest leader employees associate themselves with and the main channel of command from management to employees and from employees to management. Hence, supervisor fairness influences employees’ task performance and helping behavior (Orgambídez-Ramos and Almeida, 2017).

## Based on the Literature, We Developed the Following Hypotheses

H1: Supervisor fairness has a positive effect on work climate.
H2: Supervisor fairness has a positive effect on job satisfaction.
H3: Work climate has a positive effect on task performance.
H4: Work climate has a positive effect on helping behavior.
H5: Job satisfaction has a positive effect on task performance.
H6: Job satisfaction has a positive effect on helping behavior.
H7: Supervisor fairness has positive effects on task performance.
H8: Supervisor fairness has positive effects on helping behavior.
H9: Work climate mediates the relationship between supervisor fairness and task performance.
H10: Job satisfaction mediates the relationship between supervisor fairness and helping behavior.

## Methodology

The study adopted a quantitative approach that uses questionnaires to collect data from a cross-section of health workers within the greater Accra region of Ghana.

### Sampling and Data Collection

The researchers adopted a stratified sampling approach to select health workers in the Greater Accra region. The selection of greater Accra was based on the fact that it was the epicenter during the outbreak and most health workers have workers in hospitals that have recorded COVID-19 cases. Health facilities in major cities within the Greater Accra region were treated as stratums from which samples were selected. Questionnaires were administered by field assistance to the respondents who filled and returned them. Health workers who were active from November 2019 were targeted because the researchers perceived them to be actively engaged in COVID-19 issues during the period; most of them worked in various COVID-19 teams. Of 398 questionnaires that were administered, 362 were returned, but only 349 were fit for the analysis. Data were collected in May 2021.

### Inclusion Criteria

The bases for selection are one must be a qualified health worker employed at any hospital department, must be working in the hospitals before November 2019, must be over 18 years of age, and should be willing to participate.

### Measures

The researcher adopted or adapted the following indicators to measure the five constructs.

#### Supervisory Fairness

The study by Leung et al. (2001) suggested that supervisor fairness or justice has a universal dimension; hence, what is regarded as justice at one place is the same at other places. The researchers measured supervisors’ fairness using the phrase “my supervisor has fair policies,” and another item that is popularly used to measure supervisor fairness, which was developed by Colquitt (2001), is “My supervisor provides opportunities to appeal decisions.” We adopted the three items and added one indicator based on the COVID-19 situation (1 my supervisor has fair policies, the procedures my supervisor uses are fair, my supervisor provides opportunities to appeal decisions, and my supervisor was actively involved).

#### Job Satisfaction

Some researchers adapted the popularly used job satisfaction items developed by Spector (1985), the Job Satisfaction Survey (JSS), which was validated by Li and Huang (2017) (pay, promotion, supervision, benefits, and nature of work), while other researchers used similar tools to measure workers’ satisfaction in the healthcare sector.

#### Work Climate

Researchers adapted items from the Psychological Climate Survey (PCS; Parker et al., 2003) (role, job, organization, and supervisor).

#### Task Performance

Task performance refers to meeting the periodically assigned tasks, based on which an employee’s salary, remuneration, or other benefits are determined. The researcher adopted four instruments from Williams and Anderson (1991) and added one based on the COVID-19 situation (performs their job well, sufficiently completes assigned duties on time, performs responsibilities that match the job description, performs tasks that are expected from them, performs additional tasks as instructed).

#### Helping Behavior

Helping behavior refers to any untasked duty that an employee offers to enable coworkers to fulfill their duties in the organization’s interest. We adapted Settoon and Mossholder (2002) instruments (takes on additional responsibilities by helping coworkers when there is demand, helps coworkers with heavy workloads, performs coworkers’ duties in their absence, takes a personal interest in coworkers, demonstrates concern and politeness toward coworkers, and encourages coworkers at critical times).

#### Ethical Clearance

Ethical clearance was obtained from the Ghana Health Service in 2020 with the number GHSE/2020081123N.

#### Data Analysis

Data were analyzed using Statistical Package for Social Science (SPSS) version 21 and Moment Structure (AMOS) version 21 software. Exploratory factor analysis (EFA) and reliability tests were conducted using SPSS, which helped the researchers to measure the construct loaded with the construct and the Cronbach’s alpha of the constructs. Confirmatory factor analysis (CFA) to determine the goodness-of-fit of data was conducted using AMOS. AMOS was used due to its ability to generate results that are accurate and robust. The researchers used AMOS plugin from Gaskin and Lim (2016) to generate the construct reliability (CR) and validity of the variables. All variables loaded above 0.50; hence, no variable was deleted; this was done as recommended by Finch (2019); the loading of variables above 0.50 may be due to the adaption of variables used in several studies. Relationships between the constructs as stated on the hypotheses were tested using SPSS software.

#### Demographic Characteristics of the Samples

The demographic characteristics of respondents showed that the ages of 17% of respondents ranged from 18 to 25 years, 37.8% of them ranged from 26 to 35 years, 32.4% of them ranged from 36 to 45 years, 11.2% of them ranged from 46 to 55 years, and 0.9% of them ranged above 55 years. In terms of gender, 61.3% were women and the remaining 38.7% were men. The qualification of respondents was as follows: 8.9% had no formal education, 17.8% hold senior high school certificates, 42.4% hold diploma certificates, and 30.9 hold degrees. The number of years respondents worked at various hospitals was as follows: 2.6%: 1–5 years, 22.6%: 6–10 years, 35.0%: 10–15 years, 27.8%: above 15 years. Currently, from those holding a leadership position, 79.1% said “no,” and 20.9% responded “yes.” Departments of respondents were grouped as follows: 10.6% worked at sanitation and general stores, 55.4% were nurses, 21.8% were medical doctors, pharmacists, and physician assistants, and 12.2% did not specify their departments.

## Results

### Reliability and Validity Test Results of Variables

The EFA was completed on the variables using principal component analysis (PCA) with Varimax rotation (eigenvalue > one as cutoff). Sampling adequacy measure was conducted using Kaiser-Meyer-Olkin (KMO) which generated a sample adequacy of 0.934 and Bartlett’s test of sphericity, approx chi-square 7199.549 with the df 253 at Sig.0.000. These show the data are free from common method variance.

In Table 1 all variable factor loadings were fit as they were above 0.50 threshold, Cronbach’s alpha ranged from 0.864 to 0.977, higher than 0.7 thresholds, indicating that the data meet internal consistency level, composite reliability (CR) ranged from 0.865 to 0.978, average variance extracted (AVE) ranged from 0.616 to 0.816, both of which were higher than 0.50 threshold according to the study of Sarstedt et al. (2017) and Kadic-Maglajlic et al. (2018), which is an indication of convergent validity. Further analysis on the fitness of the model produced the following results: CMIN = 506.561, DF = 225, CMIN/DF = 3.248, CFI = 0.961, SRMR = 0.052, RMSEA = 0.064; all rated as excellent.

**TABLE 1:** Confirmatory factor analysis’s factor loading of variables according to constructs.

Variables	Codes	E	S.E.	C.R.	P	a	CR	AVE
Supervisors’ fairness	SF2	0.806				0.864	0.865	0.616
	SF3	0.788	0.066	14.452	***			
	SF4	0.75	0.074	13.644	***			
	SF1	0.795	0.071	14.598	***			
Work climate	WC3	0.912				0.896	0.922	0.695
	WC4	0.711	0.053	15.183	***			
	WC2	0.9	0.041	23.379	***			
	WC1	0.796	0.048	18.45	***			
Job satisfaction	JS5	0.954				0.977	0.978	0.816
	JS3	0.938	0.027	35.688	***			
	JS1	0.918	0.029	32.384	***			
	JS2	0.927	0.029	33.718	***			
	JS4	0.933	0.028	34.804	***			
Task performance	TP4	0.946				0.913	0.917	0.691
	TP3	0.857	0.038	23.436	***			
	TP1	0.775	0.05	18.728	***			
	TP5	0.8	0.044	19.996	***			
	TP2	0.763	0.051	18.151	***			
Helping behavior	HB3	0.923	0.029	33.167	***	0.841	0.842	0.623
	HB1	0.899	0.031	29.894	***			
	HB2	0.872	0.033	26.886	***			
	HB4	0.875	0.033	27.177	***			
	HB5	0.783	0.037	20.249	***			
	HB6	0.683	0.683	19.249	***			

α, Cronbach’s alpha; E, estimate; E.S, standardized estimate; C.R., critical ratio; p, p-value; ***p < 0.001; CR, composite reliability; AVE, average variance extracted.

### Means, Standard Deviation, and Correlation Analysis

Table 2 shows mean, SD, and inter-factor correlation analysis of demographic variables of age, gender, and qualification and the constructs of supervisors’ fairness, work climate, job satisfaction, task performance, and helping behavior. Statistically significant correlations between the variables provided initial supports for hypotheses H1, H2, H3, H4, H5, H6, H7, and H8. Though there is a statistically significant correlation between work climate and job satisfaction, that relationship is not interesting to the research objective.

**TABLE 2:** Means, SD, and inter-factor correlation.

	Variables	Mean	SD	1	2	3	4	5	6	7	8
1	Age	3.35	0.928	1							
2	Gender	1.39	0.488	−0.181**	1						
3	Qualification	2.95	0.918	0.225**	−0.294**	1					
4	SF	3.6046	0.81506	0.128*	0.088	0.088	1				
5	WC	3.6340	0.80885	0.105*	0.076	0.061	0.919**	1			
6	JS	3.5524	0.85267	0.129*	0.069	0.096	0.807**	0.839**	1		
7	TP	3.5628	0.84683	0.181**	0.093	0.095	0.894**	0.809**	0.864**	1	
8	HB	3.4064	0.65030	0.101	0.066	0.107*	0.842**	0.770**	0.803**	0.809**	1

Correlation coefficients, and p-values *p < 0.05, **p < 0.01, ***p < 0.001; SF, supervisors’ fairness; WC, work climate; JB, job satisfaction; TP, task performance; HP, helping behavior.

### Hypotheses Testing

By testing the direct and mediating effects of supervisors’ fairness, work climate, and job satisfaction on task performance and helping behavior, we performed hierarchical regression analysis on SPSS version 21 software, as shown in Table 3. The background variables (age, gender, education, duration at work, department, and title) were used as controlled variables. The results are presented in Table 3 in specific models from Model 1 to Model 6. Model 1 in Table 3 indicated that supervisors’ fairness has a positive effect on health workers’ departments. Model 2 in Table 3 shows that Supervisors’ fairness has positive effects on work climate and job satisfaction during the COVID-19 outbreak, and this indicated that hypotheses H1 and H2 were supported. Similarly, Model 3 in Table 3 shows that work climate has positive effects on task performance and helping behavior of health workers during the COVID-19 outbreak, and this also indicated that hypotheses H3 and H4 were supported. Again, Model 4 in Table 3 shows that job satisfaction has a statistically significant effect on task performance and helping behavior of health workers; hence, hypotheses H5 and H6 were equally supported. In Model 5 in Table 3, the researchers tested the direct effects of supervisors’ fairness on task performance and helping behavior of health workers, and the result shows that supervisors’ fairness has a statistically significant influence on both workers’ task performance and helping behavior. To check the influence of work climate on the relationship between supervisors’ fairness and workers’ task performance and the effects on job satisfaction on the relationship between supervisors’s fairness and workers’ helping behavior, the researchers regress supervisors’ fairness on all the four variables, as shown in Model 6 of Table 3. However, all variables were statistically significant with a significant change in R-square. There were fewer changes in the existing relationships between supervisors’ fairness and task performance, similar to the relationship between supervisors’ fairness and helping behavior; hence, the mediating effects of work climate on the relationship between supervisors’ fairness and task performance are partial; similarly, the mediating effects of job satisfaction on the relationship between supervisors’ fairness and helping behavior is partial; hence, hypotheses H9 and H10 are not wholly supported.

**TABLE 3:** Hypotheses testing with the hierarchical regression analysis to indicate the relationships between the variables and the mediating effects of work climate on the relationship between supervisors’ fairness and task performance, and job satisfaction on supervisors’ fairness and helping behavior.

Variables	Supervisor fairness (SF)	Supervisors’ fairness (SF)	Work climate (WC)	Job satisfaction (JB)	Supervisors’ fairness (SF)	Supervisors’ fairness
						
	Model 1	Model 2	Model 3	Model 4	Model 5	Model 6
	B (t)	B (t)	B (t)	B (t)	B (t)	B (t)
(Constant)	5.925***	−0.912***	1.208***	0.734***	−0.415**	−1.990*
Age	0.018 (0.210)	−0.002(−0.073)	−0.016(−0.335)	0.004 (0.103)	−0.011(0.302)	−0.005(−0.208)
Gender	0.186 (3.316)	0.028 (1.568)	0.012 (0.369)	−0.015(−0.523)	0.015 (0.616)	0.013 (0.834)
Education	0.048 (0.836)	0.010 (0.520)	−0.048(−1.445)	0.013 (0.460)	−0.104(−0.604)	0.002 (0.145)
Duration	0.108 (1.276)	0.017 (0.638)	−0.027(−0.558)	−0.030(−0.476)	−0.021(−0.588)	−0.002(−0.085)
Department	0.187**(3.226)	0.018 (0.952)	0.102 (3.073)	−0.005(−0.169)	0.060 (2.468)	0.018 (1.092)
Title	0.041 (0.766)	0.013 (0.760)	−0.001(−0.025)	−0.037(−0.1420)	−0.008(−0.378)	0.001 (0.076)
WC		0.526***(17.038)				0.420***(14.593)
JB		0.458***(14.993)				0.246***(7.377)
TP			0.531***(10.222)	0.634***(14.175)	0.616***(16.331)	0.237***(7.317)
HB			0.336***(6.618)	0.295***(6.738)	0.340***(9.215)	0.126***(4.714)
*R* ^2^	0.076	0.908***	0.703***	0.776***	0.843***	0.931***
Change in *R*^2^	0.076	0.832***	0.631***	0.727***	0.767***	0.855***
F	4.696	1544.105***	360.416**	560.743**	832.312***	1045.925**

b, Standardized beta coefficient; t-values in parenthesis; *p < 0.05, **p < 0.01, ***p < 0.001; SF, supervisors’ fairness; WC, work climate; JB, job satisfaction; TP, task performance; HP, helping behavior.

## Discussion

The initial stages of COVID-19 in the late parts of 2019 brought a lot of challenges to the health system (Onigbinde et al., 2020). With less information about the COVID-19 pandemic coupled with high death rates around the globe, leadership in health institutions’ was vital in determining directions and bringing together health workers to fight the condition (Mehrsafar et al., 2021b). Supervisors who are in the immediate top hierarchy before employees were looked up to create the needed work climate and provide the job satisfaction that motivates workers to perform their tasks and respond to other duties during critical times (Xu et al., 2020).

The study explores the vital role of supervisors’ fairness in enhancing work climate, job satisfaction, task performance, and helping behavior during the outbreak. Our findings were conclusive that supervisors’ fairness has positive effects on work climate, which means that supervisors were responsible for psychological conditions in hospitals and health centers; this finding is in relation to a study by Kim et al. (2017), which stated that, in supervisory, fairness is a key to providing a positive sense of belongingness, providing good employees’ coordination, creating trust between workers and leaders, and improving employees’ problem-solving ability. The role of supervisor fairness in creating a harmonious work climate has permanently been established in studies in both the health sector and others (Yang et al., 2009; Orgambídez-Ramos and Almeida, 2017).

Supervisors’ fairness was essential in creating job satisfaction during the COVID-19 outbreak (Alam and Parvin, 2021). Researchers who established the effects of supervisors’ fairness on job satisfaction in health delivery predated the COVID-19 outbreak (Govil et al., 2020); hence, our finding of supervisors’ fairness influencing health workers’ satisfaction during the outbreak further supports the existing studies that emphasized the essential functions of supervisor fairness in health delivery (Liu and Lin, 2021).

Supervisors’ fairness was vital in motivating employees to work in the face of critical conditions, as supervisor fairness was acknowledged as pivotal in handling the Ebola outbreak in West Africa (Buseh et al., 2015). Skills and the level of fairness demonstrated by expatriate supervisors in Sierra Leone and Liberia motivated health staff to work in light of the deadly Ebola virus (Elmahdawy et al., 2017); similarly, the impact of supervisor fairness was demonstrated in the early stages of the COVID-19 outbreak in China and other countries (Zhang Y. et al., 2021). In all cases, supervisor fairness influences health workers to work at the peril of their lives, and these resulted in a reduction in morbidity and mortality among the population (Sarkar et al., 2020).

Our finding shows that job satisfaction also influences task performance and helping behavior of health workers during the COVID-19 outbreak; similar results were found in a study that investigated the role of job satisfaction in health delivery. By any standards, satisfied health workers will deliver quality services (Vidal-Blanco et al., 2019), unless in an extreme case, as found in a few studies (Avdic et al., 2019). Assuring health workers’ promotion and increasing physical rewards such as insurance and support for their families in the case of their demise in line of duties motivated them to perform their task and help others during the scary moments of COVID-19 (Karamouzian and Madani, 2020).

Work climate determines the workplace conditions that influence workers’ behavior; it is a significant determinant of work task performance and helping behavior (Orgambídez-Ramos and Almeida, 2017). Studies have indicated that. when a good climate exists, workers work as a team rather than as individuals by complementing others’ efforts (Baltes et al., 2009). Our findings indicated that work climate has a positive effect on workers’ task performance and helping behavior. These findings are not surprising because health workers work as teams during the COVID-19 outbreak rather than as individuals who respond to health issues related to COVID-19 (Alava and Guevara, 2021). These findings are similar to the study, which concluded that team leaders’ coordinating abilities during health crises create a work environment that enables employees to work as a team, which leads to improved task performance and the support they offer each other (Anderson et al., 2019). The role of supervisors in handling the COVID-19 pandemic is worth researching globally as the outbreak brought up the creation of teams in all aspects of health delivery, with supervisors playing a key role (Mehrsafar et al., 2021a).

## Conclusion

The study shows that supervisor fairness is a key to employee productivity; it enhances job satisfaction and creates a good work climate that enables task performance and helping behavior. This was exhibited in the supervisors’ effort in leading other health workers to cure and control the COVID-19 outbreak. Health authorities must enhance the quality of supervisors’ fairness to assure employees of equity in workplaces that are vital to their ability to perform their tasks and to help others should the need be.

## Implications for Policymakers

The study revealed the importance of supervisor fairness on job satisfaction, work climate, employees’ task performance, and employees’ helping behaviors. It is prudent for leaders of health institutions to demonstrate fairness toward their employees since that will build trust and assure the employees of their efforts being recognized and rewarded duly. Sense of recognition and reward, which is driven through the fairness of supervisors, creates the climate and satisfaction that drives performance of duties and makes the employees organizational citizens.

With the above understanding, supervisors must be trained to be fair to all employees and demonstrate fairness psychologically by making workers feel that they have the support of supervisors in every appropriate action they take. They should demonstrate the supervisors’ fairness by providing equity in rewards, promotion, and other engagement. We again recommend policy change on the criteria for supervisor promotion in Ghana, which is mostly based on academic progression and long-term service to include supervisors’ exhibition of fairness skills since the fairness skills demonstrated by the supervisor are the booster for productivity and employees’ commitment.

## Data Availability Statement

Data will be made available upon reasonable demand.

## Ethics Statement

The study protocol was reviewed and approved by the Ghana Health Service Board. Informed consent was obtained during data collection.

## Author Contributions

Both authors designed the study protocol. AT performed data collection and analysis. WW did the write-up and provided funding for the research activities through his awarded funding bodies.

## Conflict of Interest

The authors declare that the research was conducted in the absence of any commercial or financial relationships that could be construed as a potential conflict of interest.

## Publisher’s Note

All claims expressed in this article are solely those of the authors and do not necessarily represent those of their affiliated organizations, or those of the publisher, the editors and the reviewers. Any product that may be evaluated in this article, or claim that may be made by its manufacturer, is not guaranteed or endorsed by the publisher.
